# Radiomics Based on Digital Mammography Helps to Identify Mammographic Masses Suspicious for Cancer

**DOI:** 10.3389/fonc.2022.843436

**Published:** 2022-04-01

**Authors:** Guangsong Wang, Dafa Shi, Qiu Guo, Haoran Zhang, Siyuan Wang, Ke Ren

**Affiliations:** ^1^ Xiang’an Hospital, Xiamen University, Xiamen, China; ^2^ Xiamen Key Laboratory of Endocrine-Related Cancer Precision Medicine, Xiamen, China

**Keywords:** breast (diagnostic), breast cancer, Mammografy, Radiomic analysis, BI-RADS (Breast imaging reporting and data system)

## Abstract

**Objectives:**

This study aims to build radiomics model of Breast Imaging Reporting and Data System (BI-RADS) category 4 and 5 mammographic masses extracted from digital mammography (DM) for mammographic masses characterization by using a sensitivity threshold similar to that of biopsy.

**Materials and Methods:**

This retrospective study included 288 female patients (age, 52.41 ± 10.31) who had BI-RADS category 4 or 5 mammographic masses with an indication for biopsy. The patients were divided into two temporal set (training set, 82 malignancies and 110 benign lesions; independent test set, 48 malignancies and 48 benign lesions). A total of 188 radiomics features were extracted from mammographic masses on the combination of craniocaudal (CC) position images and mediolateral oblique (MLO) position images. For the training set, Pearson’s correlation and the least absolute shrinkage and selection operator (LASSO) were used to select non-redundant radiomics features and useful radiomics features, respectively, and support vector machine (SVM) was applied to construct a radiomics model. The receiver operating characteristic curve (ROC) analysis was used to evaluate the classification performance of the radiomics model and to determine a threshold value with a sensitivity higher than 98% to predict the mammographic masses malignancy. For independent test set, identical threshold value was used to validate the classification performance of the radiomics model. The stability of the radiomics model was evaluated by using a fivefold cross-validation method, and two breast radiologists assessed the diagnostic agreement of the radiomics model.

**Results:**

In the training set, the radiomics model obtained an area under the receiver operating characteristic curve (AUC) of 0.934 [95% confidence intervals (95% CI), 0.898–0.971], a sensitivity of 98.8% (81/82), a threshold of 0.22, and a specificity of 60% (66/110). In the test set, the radiomics model obtained an AUC of 0.901 (95% CI, 0.835–0.961), a sensitivity of 95.8% (46/48), and a specificity of 66.7% (32/48). The radiomics model had relatively stable sensitivities in fivefold cross-validation (training set, 97.39% ± 3.9%; test set, 98.7% ± 4%).

**Conclusion:**

The radiomics method based on DM may help reduce the temporarily unnecessary invasive biopsies for benign mammographic masses over-classified in BI-RADS category 4 and 5 while providing similar diagnostic performance for malignant mammographic masses as biopsies.

## Introduction

In 2020, female breast cancer (BC) became the most common type of cancer with an estimated 2.3 million new cases (11.7%), followed by lung cancer (11.4%) (1). Treatment of BC relies on conducting an accurate diagnosis, including histological, molecular, and clinical phenotypes. Non-invasive imaging techniques such as mammography, ultrasound, and magnetic resonance (MR) are available for qualitative and quantitative analysis of BC in clinical practice. The American College of Radiology Breast Imaging Reporting and Data System (BI-RADS) is a standardized assessment structure that enables radiologists to clearly and concisely communicate results of breast imaging to referring physicians (2). In the fifth edition of the BI-RADS atlas (3), category 4 and 5 breast lesions are defined as suspicious cancerous lesions, and a biopsy is recommended for further diagnosis. Recent studies have shown that a large number of benign lesions are present in category 4 and 5 breast lesions, particularly in the mammography reporting system, exposing these patients to invasive biopsies (4–6). Depending on the technique, the sensitivity values of biopsy results ranged from 87% to >97% (7–9).

Radiomics is a high-throughput image mining technique that aims to enhance the predictive power of medical images by quantifying the morphology, intensity distribution, and texture patterns of lesions. Recent investigators have examined the role of mammography, ultrasound, and MR radiomics in the prediction of molecular subtypes (10–12), lymph node metastasis (13–15), response to neoadjuvant chemotherapy (16–18), recurrence risk (19, 20), and disease-free survival (21, 22) of BC. However, with the initiative of precision medicine (23–25), the reduction in overdiagnosis and overtreatment of breast lesions through non-invasive radiomics method is also a topic worth investigating.

There are four main findings of breast lesions on diagnostic mammography images: masses, calcifications, architectural distortion, and asymmetries. One large sample study (26) showed that BC most often presented as mass at 56%, followed by calcifications at 29%, asymmetry at 12%, and architectural deformities at 4%, and another small sample study (27) suggest that approximately 50% of breast lesions presenting as a mass were ultimately confirmed benign lesions. In addition, mass may be the only finding or one of the combined findings of breast lesions in mammography (we defined these masses as mammographic masses). Although experienced radiologists have a high diagnostic accuracy in identifying benign and malignant mammographic masses, less experienced radiologists sometimes make excess errors (28) such as benign mammographic masses are over-classified as BIRADS category 4 or even 5.

A previous study has shown that combining both craniocaudal (CC) and mediolateral oblique (MLO) positions radiomics data had good classification performance between HER2-enriched BC and non-HER2-enriched BC (11). Here, we combined both CC and MLO positions radiomics data aimed to explore a model with a sensitivity more than 98% for the characterization of BI-RADS category 4 and 5 mammographic masses, thereby achieving a reduction in biopsies of benign lesions at a very low rate of missed malignant lesions.

## Materials and Methods

### Patients

This retrospective study was granted approval by the local institutional board, and written informed consent was waived. A total of 288 patients’ clinical and mammographic images data were included in this study from December 2018 to February 2021. The inclusion criteria were as follows: (a) patients who had suspected breast tumor accepted mammography and (b) patients with mass as defined by BI-RADS mammography lexicon (occupancy structures with protrude outward in contour on both CC and MLO position images) and classified in category 4 or 5. The exclusion criteria were as follows: (a) patients without a clear benign or malignant pathological result; (b) patients who had multifocal or bilateral mammographic masses; (c) patients accepted biopsy before mammography examination; (d) patients underwent any treatment before mammography screening, including surgery, chemotherapy or radiotherapy, and anti-HER2 therapy.

### Imaging and Saving Acquisition

All patients were examined with a GE Senographe Essential (GE Medical Systems, Waukesha, WI), and all mammographic images were saved at 12-bit quantization level and 100-μm pixel size. The mammographic images were not further processed or normalized (29, 30).

### Radiomics Analysis of Mammographic Masses

**Figure 1:**
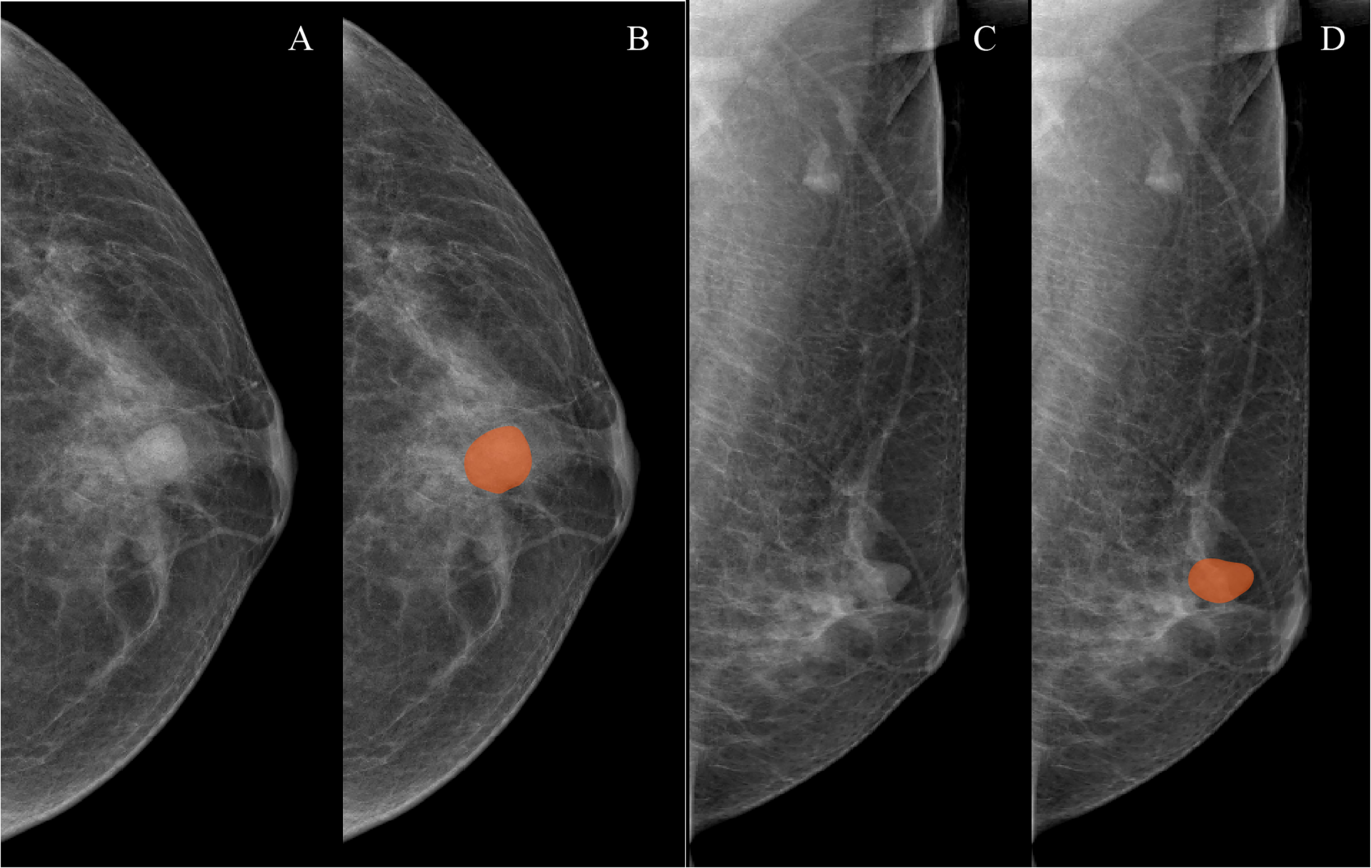
Examples of mammographic masses masking on digital mammography images. **(A, C)** Craniocaudal (CC) position images and mediolateral oblique position (MLO) images, respectively. **(B, D)** Manually drawn areas of mammographic masses masking on CC and MLO images, respectively.

Both CC and MLO position images of all patients were used to conduct mammographic mass masking, and the CC radiomics features and MLO radiomics features were extracted as separate features. Two breast radiologists (radiologist 1, 4 years’ experience; radiologist 2, 10 years’ experience) who were blinded to the pathological results manually masked the masses in 3Dslicer 4.10.2 (www.slicer.org) (**Figure 1**). A total of 188 radiomics features were extracted from mammographic masses by using the Pyradiomics python package, including 4 shape features, 34 density features, and 150 texture features. Shape features were used to quantify the size and regularity of the mammographic masses, including maximum two-dimensional (2D) diameter and perimeter to surface ratio, with a lower value of perimeter-to-surface ratio indicating a more regular mammographic mass. First-order features refer to radiologically relevant information about the density of mammographic masses such as mean value and kurtosis. Texture features were calculated based on gray-level co-occurrence matrix, gray-level dependence matrix, gray-level run-length matrix, gray-level size zone matrix, and neighborhood gray-tone difference matrix, which were used to quantify the randomness, correlations, variation, homogeneity, and heterogeneity of mammographic masses. The detailed formulae for the calculation of the radiomics features can be found here (31), and the data from radiologist 1 were used to build a radiomics model.

### Patients Grouping and Feature Selection

The patients were divided into two temporal sets based on the order in which they accepted their mammography examinations. The training set consisted of the first two-thirds of patients, and the independent test set comprised the last one-third of patients. In order to avoid some potential bias such as model over-fitting, we applied Pearson’s correlation and the least absolute shrinkage and selection operator (LASSO) regression to screen out non-redundant and useful radiomics features in the training set, respectively. For the Pearson’s correlation method, each radiomics feature generated 187 correlation coefficients and 1 corresponding mean absolute correlation coefficient. If two radiomics features had a coefficient exceeding 0.8, the radiomics feature with the larger mean absolute correlation coefficient was deleted. This was implemented in R software version 4.0.1 with package “caret.” For the LASSO regression (alpha=1, no elastic net), a 10-fold cross-validation method with 1 standard error of the minimum mean-square error criteria was used to select radiomics features (32), and corresponding λ values were also be calculated. In this study, the radiomics features with non-zero coefficient at the suitable value of parameter λ were determined as useful radiomics features. This was implemented in R software version 4.0.1 with “glmnet” package.

### Radiomics Model Construction and Testing

**Figure 2:**
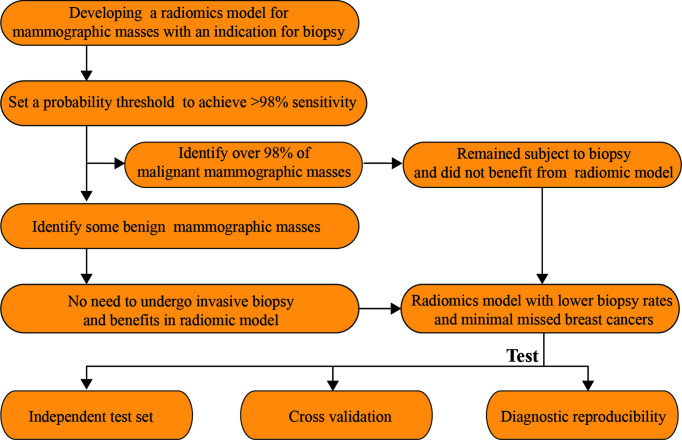
Workflows of this study.

The malignant mammographic masses were coded as 1, and the benign mammographic masses were coded as 0. The support vector machines (SVMs) with linear kernel (output predicted probability and other parameters are default parameters) were used to construct a radiomics model in this study because of popularity and efficiency in BC (33). This was done in R software version 4.0.1 with “e1071” package. The useful radiomics features were used to construct a radiomics model in the training set for distinguishing between benign and malignant mammographic masses. The receiver operating characteristic curve (ROC) analysis was used to evaluate the classification performance of the radiomics model, including the area under the receiver operating characteristic curve (AUC), the probability threshold value (cut-point) of higher than 98% sensitivity, and corresponding specificity (5). The independent test set was used to test the results of the training set by using the “predict” function, and the probability threshold value was also tested. The ROC analysis was implemented in R software version 4.0.1 with package “pROC.” The workflow of this study is reported in **Figure 2**.

### Relative Importance of Useful Radiomics Features

The magnitude of the coefficients in the LASSO algorithm were used to measure the relative weight of useful features as previously described (34, 35). Furthermore, the useful features were grouped by category, such as shape feature group, first-order feature group, and texture feature group, which were added sequentially to the final SVM model, and the AUC of each addition was calculated to assess whether all three groups of useful features contributed to the model.

### Radiomics Model Stability

In this study, a fivefold cross-validation method was used to evaluate the stability of radiomics model. In order not to break the concealment of the test set data, a fivefold cross-validation method was performed separately in the training and test sets. Specifically, the training and test sets were divided into 10 subsets each by fivefold cross-validation method. The “predict” function of R was used to test the diagnostic performance of the radiomics model in these subsets, and the mean and standard deviation of AUCs, sensitivities, and specificities were calculated to measure the stability of the radiomics model. The fivefold cross-validation method was performed in R software version 4.0.1 with package “caret.”

### Reproducibility Assessment

The intra-class correlation coefficients (ICCs) of interobserver (radiologist 1 vs. radiologist 2) were calculated to evaluate the reproducibility of the radiomics features extraction. All data from radiologists 1 and 2 were used as separate test sets so that a kappa value for both radiologists could be calculated to assess the diagnostic reproducibility of the radiomics model.

### Statistical Analysis

All statistical analyses were performed in R software version 4.0.1. All ROC analysis were based on package “pROC,” and the differences of AUC were calculated on Delong’s test. All confidence intervals (CI) were derived from 1,000 bootstrap replicates. All statistical tests were two-sided, and the Bonferroni’s method was used to adjust for multiple comparisons.

## Results

### Clinical Data of Patients

This study included 288 female patients (age, 52.41± 10.31) with solitary BI-RADS category 4 or 5 mammographic masses, including 130 cases of malignant mammographic masses and 158 cases of benign mammographic masses. The malignant mammographic masses include invasive ductal carcinoma (*n*=51), invasive lobular carcinoma (*n*=37), mucinous carcinoma (*n*=22), and ductal carcinoma *in situ* (*n*=20), while benign mammographic masses include fibroadenomas (*n*=87), adenosis (*n*=38), and hyperplasia (*n*=33).

### Patients Grouping and Feature Selection

**Figure 3:**
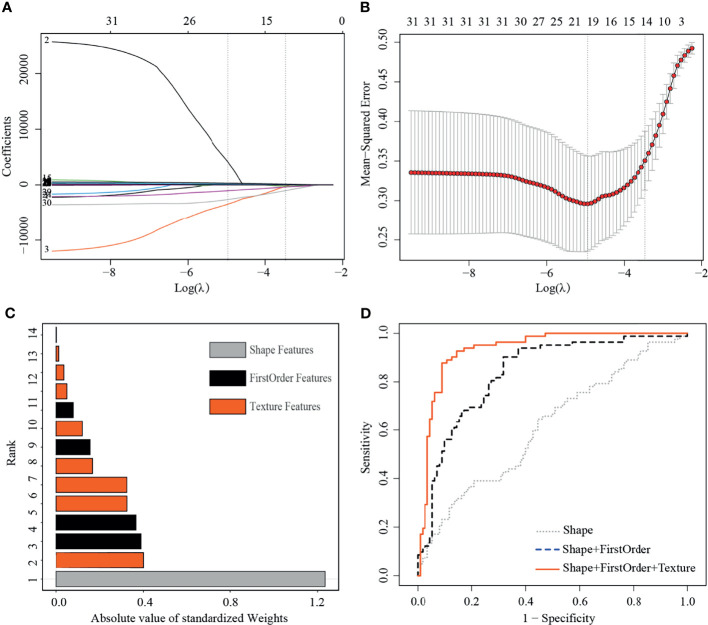
Least absolute shrinkage and selection operator (LASSO) selection process, absolute values of weights, and receiver operating characteristic curves (ROC) of 14 useful radiomics features in training set. **(A)** LASSO coefficient profiles of the 32 non-redundant features. The y-axis represents coefficient of each feature. The optimal value of λ was 0.0345, and the optimal log(λ) was −3.37, resulting in 14 non-zero coefficients. **(B)** Mean square error path using tenfold cross-validation. **(C)** Absolute value of weights generated by the LASSO algorithms for the optimal log(λ) value. **(D)** The ROC curves for a combination of shape feature, first order features, and texture features.

The training set included 82 malignant and 110 benign mammographic masses; the independent test set consisted of 48 malignant and 48 benign mammographic masses. Baseline characteristics of study population in training and test sets are reported in **Table 1**, including age, mass size, mass shape, mass margin, breast density, and BI-RADS category. The Pearson’s correlation method screened out 32 non-redundant radiomics features (**Supplementary Figure S1**), and the LASSO method further selected 14 useful radiomics features (**Figures 3A, B**).

**Table 1:** Baseline characteristics of study population in training and test sets.

Characters	Training set			Test set		
	Malignant(n= 82)	Benign(n= 110)	*p*-value	Malignant(n= 48)	Benign(n= 48)	*p*-value
Age (mean ± SD, years)	54.2 ± 10.94	50.38 ± 9.71	0.013	56.66 ± 10.33	49.75 ± 8.76	<0.001
Size (mean ± SD, cm)	1.5 ± 0.51	1.34 ± 0.42	0.021	1.69 ± 0.58	1.3 ± 0.42	<0.001
Shape			0.002			<0.001
Round or oval	10 (12.2%)	34 (30.9%)		5 (10.4%)	20 (41.7%)	
Irregular	72 (87.8%)	76 (69.1%)		43 (89.6%)	28 (58.3%)	
Margin			<0.001			<0.001
Circumscribed	5 (6.1%)	16 (14.5%)		3 (6.3%)	12 (25%)	
Ill-defined	26 (31.7%)	82 (74.5%)		22 (45.8%)	33 (68.8%)	
Spiculated	51 (62.2%)	12 (10.9%)		23 (47.9%)	3 (6.3%)	
Breast density			0.169			0.094
Entirely fatty	7 (8.5%)	15 (13.6%)		4 (8.3%)	7 (14.6%)	
Scattered fibroglandular	37 (45.1%)	58 (52.7%)		22 (45.8%)	29 (60.4%)	
Heterogeneously dense	38 (46.3%)	37 (33.6%)		22 (45.8%)	12 (25%)	
Extremely dense	…	…				
BI-RADS category			<0.001			0.031
4	64 (78%)	105 (95.5%)		38 (79.2%)	46 (95.8%)	
5	18 (22%)	5 (4.5%)		10 (20.8%)	2 (4.2%)	

Student’s t-test for normally distributed continuous variable (age and size); Pearson’s chi-square test for categorical variables (shape, margin, breast density, BI-RADS categories, and pathological results).

SD, standard deviation; BI-RADS, breast imaging reporting and data system.

### Radiomics Model Construction and Testing

**Figure 4:**
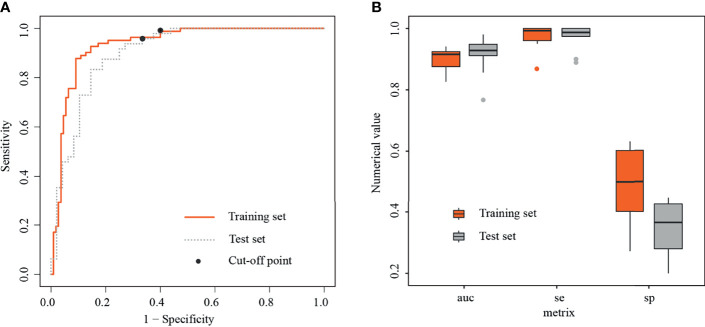
Receiver operating characteristic curves and boxplots in training and test sets. **(A)** Receiver operating characteristic curves (ROC) in training and test sets, with black dots representing threshold=0.22. **(B)** Boxplots of area under the curves (AUCs), sensitivities, and specificities generated by fivefold cross-validation in training and test sets, respectively.

In the training set, the radiomics model obtained an AUC of 0.934 (95% CI, 0.898–0.971), a threshold of 0.22, a sensitivity of 98.8% [81/82], and a specificity of 60% [66/110]. In the test set, the radiomics model obtained an AUC of 0.901 (95% CI, 0.835–0.961), a sensitivity of 95.8% [46/48], and a specificity of 66.7% [32/48] (**Figure 4A**).

### Relative Importance of Useful Radiomics Features

For the useful single radiomics feature, perimeter-to-surface ratio from shape features had the largest absolute weight value of 1.234, followed by coarseness from texture features of 0.4 and mean from first-order features of 0.389 (**Figure 3C**, details in **Table 2**). For useful single-category radiomics features, texture feature group obtained the largest absolute weight value of 1.435, followed by shape feature group of 1.234, and lastly by first-order feature group of 0.96 (**Figure 3C**, details in **Table 2**). When shape feature group, first-order feature group, and texture feature group were added sequentially to the final SVM model, ROC analysis showed a significant increase in AUC for each addition (shape feature group, AUC=0.613; shape feature group plus first-order feature group, AUC=0.835; first-order feature group plus and first-order feature group plus texture feature group, AUC=0.934; p<0.001 for each addition) (**Figure 3D**, details in **Table 3**).

**Table 2:** Absolute value of weights of selected useful radiomics features in training set.

Feature category	Feature name	Absolute weights
Shape	MLO/Sphericity	1.234
FirstOrder	CC/Mean	0.389
	MLO/Uniformity	0.366
	CC/Skewness	0.156
	MLO/Mean	0.079
Texture	MLO/NGTDM Coarseness	0.4
	MLO/GLSZM GrayLevelNonUniformity	0.325
	CC/GLDM SmallDependenceHighGrayLevelEmphasis	0.324
	MLO/GLDM LargeDependenceLowGrayLevelEmphasis	0.167
	CC/NGTDM Coarseness	0.12
	CC/NGTDM Busyness	0.05
	MLO/GLCM InverseDifferenceNormalized	0.036
	CC/GLSZM ZoneVariance	0.012
	CC/GLCM ClusterProminence	0.001

CC, craniocaudal; MLO, mediolateral oblique; NGTDM, neighborhood gray-tone difference matrix; GLSZM, gray-level size zone matrix; GLDM, gray-level dependence matrix; GLCM, gray-level co-occurrence matrix.

**Table 3:** Classification performance of selected shape feature, first order features, and texture features in classifying malignancies and benign lesions in the training set.

Features	AUC^*^	*p-*value^†^
Shape	0.613 (0.528, 0.695)	…
Shape+FirstOrder	0.835 (0.774, 0.888)	…
Shape+FirstOrder+Texture	0.934 (0.898, 0.968)	…
Shape vs (Shape+FirstOrder)	…	<0.001 (0.017)
Shape vs (Shape+FirstOrder+Texture)	…	<0.001 (0.017)
(Shape+FirstOrder) vs (Shape+FirstOrder+Texture)	…	<0.001 (0.017)

^*^Numbers in parentheses are 95% confidence intervals.

^†^Numbers in parentheses are the significance level.

AUC, area under the receiver operating characteristic curve.

### Radiomics Model Stability

In the training set, the average AUC was 0.9 ± 0.038, average sensitivity was 97.39% ± 3.9%, and average specificity was 50% ± 12.5%. In the test set, the average AUC was 0.915 ± 0.062, average sensitivity was 98.7% ± 4%, and average specificity was 36.7% ± 8.6% (**Figure 4B**, details in **Table 4** and **Supplementary Figure S2**).

**Table 4:** Classification performance of radiomics model in fivefold cross-validation.

Data set	Fivefold CV	Pathology results			AUC	Sensitivity (%)	Specificity (%)
		Malignant	Benign	p			
Training set	–Fold 1	67	87	0.653	0.941 (0.898, 0.977)	98.5 [66/67]	50.6 [44/87]
	Fold 1	15	23		0.910 (0.806, 0.997)	100 [15/15]	39.1 [9/23]
	–Fold 2	65	88	0.901	0.927 (0.881, 0.965)	100 [65/65]	30.7 [27/88]
	Fold 2	17	22		0.912 (0.786, 1)	100 [17/17]	27.2 [6/22]
	–Fold 3	62	91	0.302	0.926 (0.878, 0.967)	95.2 [59/62]	58.2 [53/91]
	Fold 3	20	19		0.856 (0.713, 0.966)	95 [19/20]	63.2 [12/19]
	–Fold 4	67	87	0.653	0.920 (0.871, 0.962)	98.5 [66/67]	63.2 [55/87]
	Fold 4	15	23		0.826 (0.672, 0.954)	100 [15/15]	43.5 [10/23]
	–Fold 5	67	87	0.653	0.919 (0.872, 0.960)	100 [67/67]	49.4 [43/87]
	Fold 5	15	23		0.864 (0.742, 0.965)	86.7 [13/15]	60.9 [14/23]
	*Mean± SD*	*…*	*…*		*0.9 ± 0.038*	*97.39 ± 3.9*	*50 ± 12.5*
Test set	–Fold 1	38	39	0.798	0.919 (0.846, 0.976)	97.4 [37/38]	33.3 [13/39]
	Fold 1	10	9		0.967 (0.867, 1)	100 [10/10]	44.4 [4/9]
	–Fold 2	38	38	1	0.912 (0.837, 0.968)	100 [38/38]	23.7 [9/38]
	Fold 2	10	10		0.980 (0.920, 1)	100 [10/10]	20 [2/10]
	–Fold 3	39	38	0.798	0.947 (0.883, 0.994)	97.4 [38/39]	44.7 [17/38]
	Fold 3	9	10		0.856 (0.667, 1)	100 [9/9]	40 [4/10]
	–Fold 4	39	38	0.798	0.937 (0.879, 0.981)	100 [39/39]	26.3 [10/38]
	Fold 4	9	10		0.911 (0.688, 1)	88.9 [8/9]	40 [4/10]
	–Fold 5	38	39	0.798	0.949 (0.887, 0.993)	97.4 [37/38]	43.6 [17/39]
	Fold 5	10	9		0.767 (0.500, 1)	90 [9/10]	33.3 [3/9]
	*Mean± SD*	*…*	*…*		*0.915 ± 0.062*	*98.7 ± 4*	*36.7 ± 8.6*

CV, cross validation; AUC, area under the receiver operating characteristic curve; SD, standard deviation.

### Reproducibility Assessment

Of the 188 extracted radiomics feature, more than 95% [180/188] radiomics features obtained good reproducibility (ICC>0.75) between radiologist 1 and radiologist 2. The radiomics model obtained good agreement between radiologist 1 and radiologist 2 (Cohen’s kappa=0.748; 95% CI, 0.67–0.825; p<0.001).

## Discussion

In this study, we built a radiomics model with similar sensitivity to biopsy for predicting malignancy of BI-RADS category 4 and 5 mammographic masses by using the combination of CC and MLO position images from DM. In both training and test sets, the radiomics model obtained specificity by over 60% while preserving sensitivity more than 95.8%. Both AUC and sensitivity were relatively stable, while the specificity was not so stable. These experimental results suggest that the non-invasive imaging radiomics method could achieve similar sensitivity to biopsy while avoiding some benign mammographic masses to undergo unnecessary invasive biopsy.

We are aware of several papers that use mammographic radiomics data to differentiate between benign and malignant breast lesions (30, 36–38), and we are aware that several of these papers incorporated morphological features (36, 37), and several that did not (30, 38). However, most breast lesions that present alone as calcification, architectural distortion, or asymmetries tend to lack distinct grayscale contrast boundaries, which compromises the masking of tumors. Besides, non-invasive imaging techniques with similar biopsy sensitivity are important for differentiating between benign and malignant BI-RADS category 4 and 5 breast lesions for which invasive biopsy was already indicated. To the best of our knowledge, this is the first study to use mammographic radiomics data to predict the malignancy of BI-RADS category 4 and 5 mammographic masses with a sensitivity similar to that of a biopsy, and we believe that the radiomics model is more limited but more relevant.

When using radiomics data to predict the malignancy of breast lesions, commonly used mammography images include CC position alone (37, 39), mixed CC and MLO position (30, 36), and combination of CC position and MLO position (40, 41). Gupta et al. demonstrated that the corresponding first-order and texture features of mammographic masses between CC position and MLO position were not strongly correlated (42), suggesting that the inclusion of first-order and texture features from multiple mammographic positions may impact the accuracy of diagnosis of mammographic masses. Ma et al. has shown that by combining both CC and MLO position, radiomics data had higher classification performance between HER2-enriched BC and non-HER2-enriched BC than using CC position alone and MLO position alone (11). Hence, we applied both CC and MLO position radiomics data to predict the malignancy of mammographic masses suspicious for cancer, and the results showed that this method had good classification performance.

Recent studies have shown that the use of a single random training–test set split may lead to unreliable results in small sample radiomics machine learning studies (43). In our study, we divided the training and test sets based on the chronological order in which patients underwent mammography examinations, and we performed random fivefold cross-validation in training and test sets, respectively, to assess the stability of the radiomics model. Although the specificity gives a large error (12.5%, 8.6%), the AUC (0.038, 0.062) and sensitivity (3.9%, 4%) were relatively stable. This is consistent with previous findings that cross-validation may lead to large error bars for small sample sizes (44).

**Figure 5:**
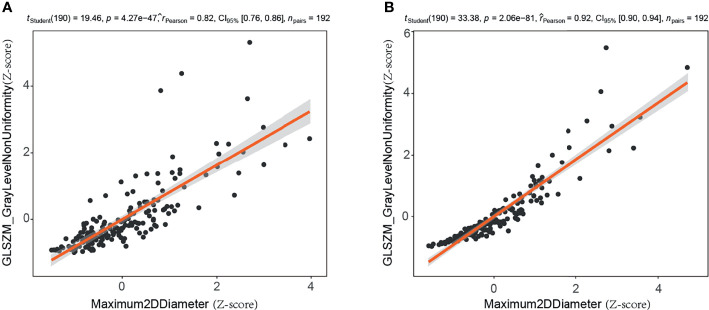
Correlation analysis of maximum two-dimensional diameter and gray level non-uniformity (gray-level size zone matrix based) in training set. **(A)** Craniocaudal (CC) position images; **(B)** mediolateral oblique (MLO) position images. 2D, two-dimensional; GLSZM, gray-level size zone matrix.

Of the 188 mammographic radiomics features, the feature selection method screened out 14 useful features in the training set (n=192, ratio 14:1). As suggested by Gillies et al., each radiomics feature requires at least 10 samples to support in a classifier (45). Previous publication has shown that maximum 2D diameter is a useful feature for predicting BC (37). In this study, we found a significant positive correlation between maximum 2D diameter and gray-level size zone matrix-based gray level non-uniformity in the CC position images (r=0.82, p<0.001) and MLO position images (r=0.924, p<0.001) of the training set (**Figure 5**). Thus, the maximum 2D diameter feature was removed when filtering features in the Pearson’s correlation analysis. It is worth noting that although the size feature was not included in our radiomics model, we do not consider it unimportant in predicting malignancy of mammographic masses suspicious for cancer.

**Figure 6:**
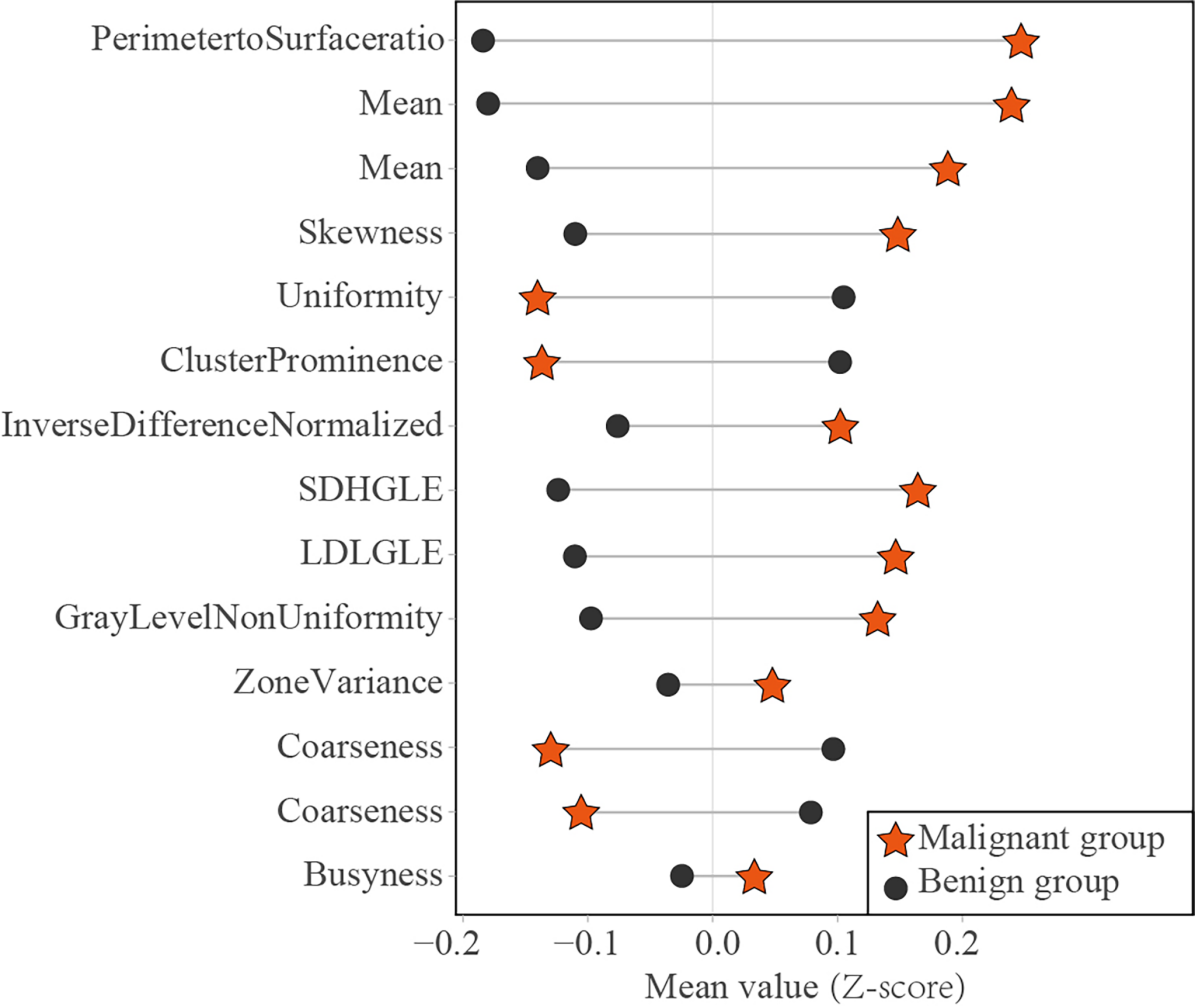
Dumbbell diagram of the mean value of 14 useful radiomics features between the malignant and benign groups in the training set. SDHGLE, small-dependence high gray-level emphasis; LDLGLE, large-dependence low gray-level emphasis.

Of the 14 useful mammographic radiomics features, perimeter-to-surface ratio had the greatest weight value of 1.234, suggesting that morphological features remain important in identifying benign lesions or malignancies for mammographic masses suspicious for cancer. The mean density obtained moderate weight values of 0.389 and 0.079, which was higher in the malignant group than in the benign group, indicating that the malignant mammographic masses tend to be higher density than benign mammographic masses. In addition, the malignant group had higher value for inverse difference normalized, zone variance, gray level non-uniformity, and busyness, and lower value for uniformity, skewness, coarseness, small-dependence high gray-level emphasis, and large-dependence low-gray level emphasis, indicating more heterogeneity of malignant mammographic masses in the density and texture patterns (31) (**Figure 6**). This experimental result is consistent with the pathological fact that malignant tumors tend to be heterogeneous, while benign lesions tend to be homogeneous (46).

Admittedly, our study has several limitations. First, mammographic radiomics data for this study were collected from a single center with a limited number of participants, and further multicenter testing is needed. Second, the mammographic masses were masked by manual method; however, good inter-observer reproducibility was obtained in feature extraction and model diagnosis. Some publications indicate that semi-automatic segmentation method had higher inter-observer reproducibility (47, 48). Further work is needed to construct a semi-automatic segmentation method for mammographic masses. Finally, the radiomics model was constructed by 2D images, which may lose some important information of mammographic masses. However, some publications showed that 2D radiomics features had higher classification performance than 3D radiomics features in lung cancer (47, 48).

In conclusion, a mammographic radiomics model combining both CC and MLO position images had excellent sensitivity and moderate specificity in differentiating malignancies and benign lesions for BI-RADS category 4 and 5 mammographic masses. It may help reduce the temporarily unnecessary invasive biopsies for benign mammographic masses while providing similar diagnostic performance for malignant mammographic masses as biopsies.

## Data Availability Statement

The raw data supporting the conclusions of this article will be made available by the authors, without undue reservation.

## Ethics Statement

The studies involving human participants were reviewed and approved by the Ethics Committee of Xiang’an Hospital of Xiamen University. Written informed consent for participation was not required for this study in accordance with the national legislation and the institutional requirements.

## Author Contributions

Study concept and design: GW and DS. Acquisition of data: HZ, QG, and SW. Analysis of data: GW, DS. Drafting of the manuscript: GW, DS, HZ, and SW. Critical revision: QG and KR. Statistical analysis: GW and DS. Study supervision: QG and KR. All authors contributed to the article and approved the submitted version.

## Funding

This work was supported by the Scientific Research Foundation for Advanced Talents, Xiang’an Hospital of Xiamen University (no. PM201809170011).

## Conflict of Interest

The authors declare that the research was conducted in the absence of any commercial or financial relationships that could be construed as a potential conflict of interest.

## Publisher’s Note

All claims expressed in this article are solely those of the authors and do not necessarily represent those of their affiliated organizations, or those of the publisher, the editors and the reviewers. Any product that may be evaluated in this article, or claim that may be made by its manufacturer, is not guaranteed or endorsed by the publisher.
