# Supplementary material for: Radiomics Based on Digital Mammography Helps to Identify Mammographic Masses Suspicious for Cancer
Source: Front Oncol. 2022 Apr 1;12:843436. doi: 10.3389/fonc.2022.843436 (PMC9012139; doi:10.3389/fonc.2022.843436)
Supplement: Supplementary file 1 [file DataSheet_1.docx]

Supplementary Material

## Supplementary Figures

**
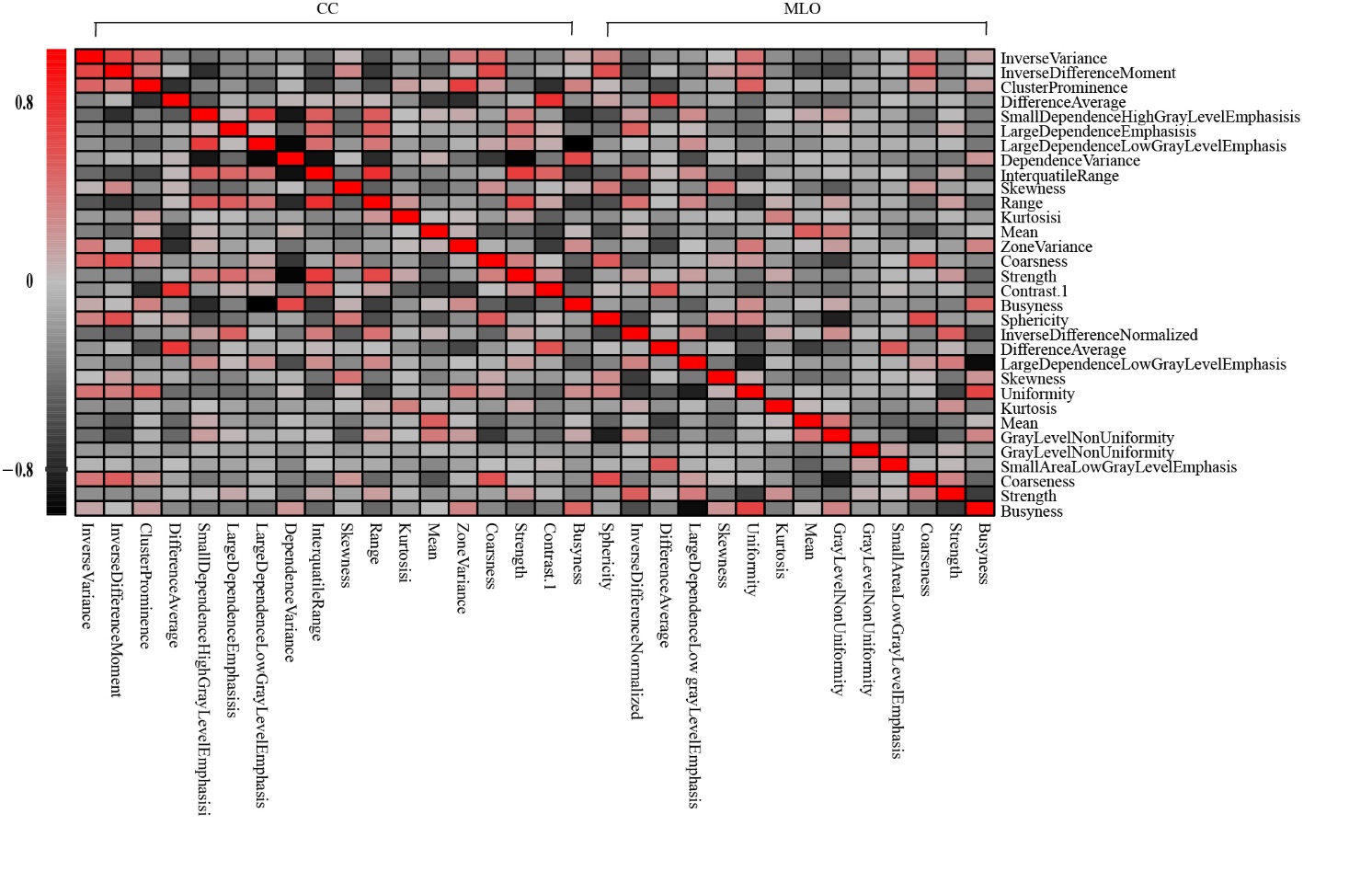
**

**Supplementary Figure 1.** Heat map of correlation coefficients of 32 non-redundant radiomics features selected by Pearson’s method. A redder and blacker color indicates a higher absolute value of the correlation coefficient. CC, craniocaudal; MLO, mediolateral oblique.


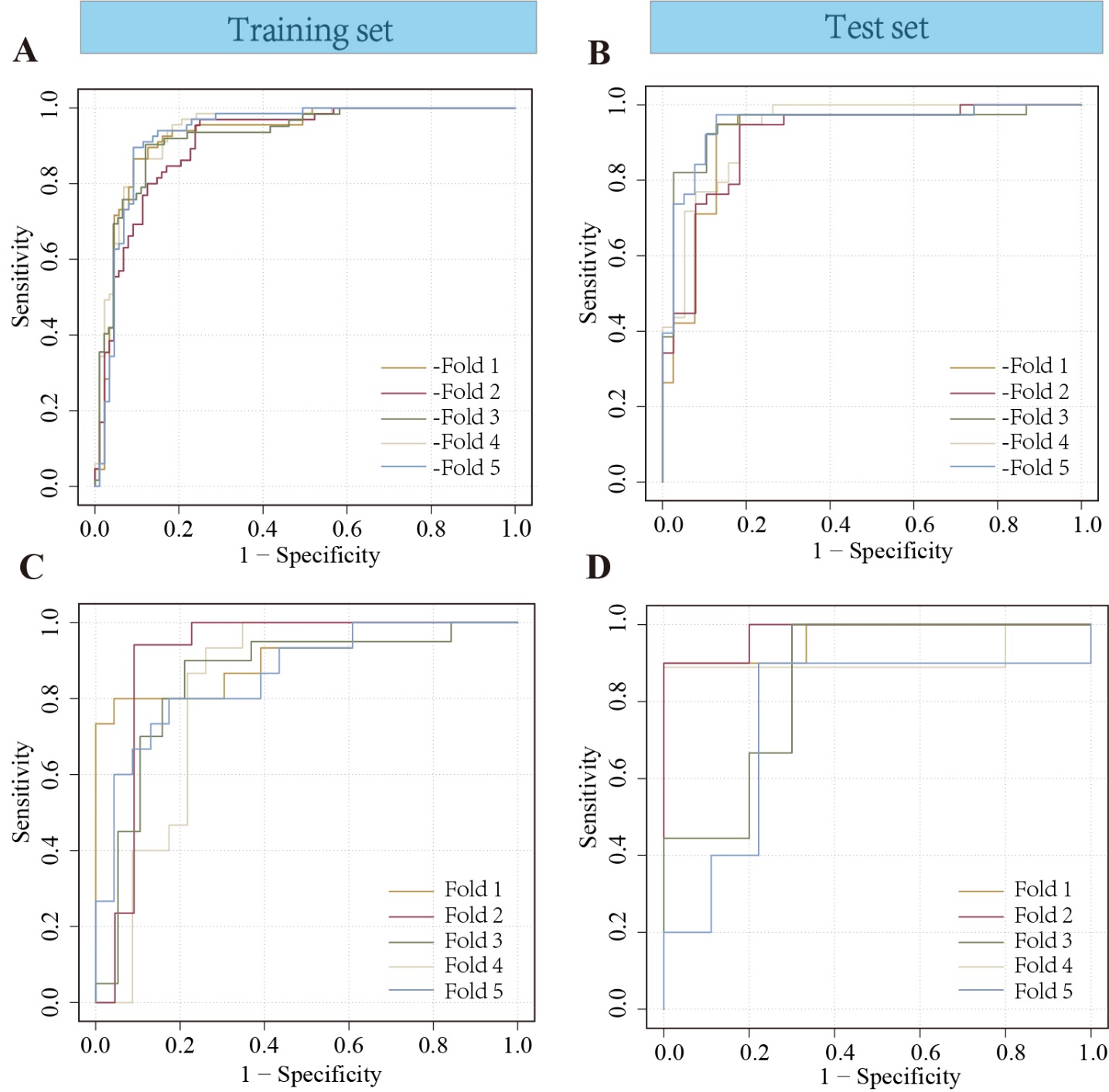


**Supplementary Figure 2.** The ROC curves of 5-fold cross validation method in training set and test set. **(A, C)** Training set. **(B, D)** Test set.
